# Implantable Cardioverter-Defibrillator Therapy in Patients With Transposition of the Great Arteries: A Systematic Review of the Literature

**DOI:** 10.1016/j.cjcpc.2025.04.006

**Published:** 2025-04-24

**Authors:** Jessica Victoria Yao, Ximena Cid-Serra, Karrar Albadosh, Andrew Browne, Anastasia D. Egorova, Magalie Ladouceur, Craig Broberg, Muhammad A. Nizam, Paul Khairy, Gareth J. Padfield, Jeremy P. Moore, Geetha Kandavello, Sushma Reddy, Gruschen Veldtman, Dominica Zentner

**Affiliations:** a Department of Medicine, Dentistry and Health Sciences, the University of Melbourne, Parkville, Victoria, Australia; b Department of Cardiology, the Royal Melbourne Hospital, Parkville, Victoria, Australia; c Department of Surgery, the University of Melbourne, Parkville, Victoria, Australia; d Department of General Medicine, the Royal Melbourne Hospital, Parkville, Victoria, Australia; e Department of Cardiology, Golden Jubilee National Hospital, Scotland, United Kingdom; f Nuffield Department of Population Health, University of Oxford, Oxford, United Kingdom; g Department of Cardiology, Leiden University Medical Centre, Leiden, the Netherlands; h Center for Congenital Heart Disease Amsterdam-Leiden (CAHAL), Leiden, the Netherlands; i Department of Cardiology, University Hospital of Geneva, Geneva, Switzerland; j Department of Cardiology, University Hospital of Lausanne, Lausanne, Switzerland; k Department of Cardiology, Oregon Health and Science University, Portland, Oregon, USA; l Department of Internal Medicine, Trinity Health Livonia Hospital, Livonia, Michigan, USA; m Electrophysiology Service and Adult Congenital Centre, Montreal Heart Institute, University of Montreal, Montreal, Québec, Canada; n University of California Los Angeles (UCLA) Cardiac Arrhythmia Center, UCLA Health System, Los Angeles, California, USA; o Ahmanson/UCLA Adult Congenital Heart Disease Centre, Department of Medicine, Division of Cardiology, Los Angeles, California, USA; p Pediatric and Congenital Heart Center, National Heart Institute (Institut Jantung Negara), Kuala Lumpur, Malaysia; q Department of Cardiology, Lucile Packard Children’s Hospital Stanford, Palo Alto, California, USA; r Helen DeVos Childrens Hospital, Corewell Health, Grand Rapids, Michigan, USA

## Abstract

**Background:**

Patients with a systemic right ventricle (sRV) due to transposition of the great arteries are known to have a particularly high risk of sudden cardiac death. Current guidelines issue a weak recommendation to consider primary prevention implantable cardioverter-defibrillator (ICD) implantation in patients with severe sRV dysfunction. This systematic review aims to ascertain factors that are associated with appropriate ICD therapy in patients with an sRV and primary prevention ICD, so that we can further refine selection criteria for implantation in this population.

**Methods:**

A systematic search of MEDLINE and Embase was performed to identify all studies that explored ICD therapies and associated clinical characteristics in patients with an sRV and primary prevention ICD from the inception of the databases until February 14, 2023.

**Results:**

A total of 11 articles were included in the final analysis. Among those with a primary prevention ICD, 23 (9.1%) had appropriate ICD therapies and 48 (19%) received inappropriate ICD therapies. Among those with appropriate ICD therapies, the most common reason for implantation was sRV dysfunction, followed by nonsustained ventricular tachycardia and ventricular tachycardia on a Holter monitor. Among those with a secondary prevention ICD, 15 (34.9%) received appropriate ICD therapies and 5 (11%) had inappropriate ICD therapies. Most inappropriate therapies were due to atrial tachyarrhythmias.

**Conclusions:**

sRV dysfunction was the most consistently reported risk factor for appropriate ICD therapy in our review. Effective treatment of atrial tachyarrhythmias remains a priority. Larger scale studies are required to develop and validate risk calculation in this population.

## Résumé

**Contexte:**

Les patients présentant un ventricule droit systémique (VDs) dû à la transposition des grosses artères sont réputés courir un risque particulièrement élevé de mort subite d’origine cardiaque. Les lignes directrices actuelles contiennent une recommandation de bas grade concernant l’utilisation d’un défibrillateur cardioverteur implantable (DCI) en prévention primaire chez les patients présentant un VDs associé à une dysfonction sévère. Cette revue systématique vise à établir les facteurs déterminant l’utilisation appropriée d’un DCI chez les patients présentant un VDs et l’utilisation d’un DCI en prévention primaire afin de pouvoir affiner davantage les critères de sélection en vue de l’implantation de ce dispositif dans cette population.

**Méthodologie:**

Nous avons effectué une recherche systématique dans les bases de données MEDLINE et Embase pour recenser toutes les études ayant évalué l’implantation d’un DCI et les caractéristiques cliniques associées chez les patients présentant un VDs ayant reçu un DCI en prévention primaire, depuis la création des bases de données jusqu’au 14 février 2023.

**Résultats:**

Au total, 11 articles ont été inclus dans l’analyse finale. Parmi les personnes ayant reçu un DCI en prévention primaire, 23 (9,1 %) avaient reçu un traitement approprié et 48 (19 %), un traitement inapproprié. Parmi les patients ayant reçu un traitement approprié, la raison la plus fréquente de l’implantation était la dysfonction associée au VDs, suivi de la tachycardie ventriculaire non soutenue et de la tachycardie ventriculaire sous moniteur Holter. Parmi les personnes ayant reçu un DCI en prévention secondaire, 15 (34,9 %) avaient reçu un traitement approprié et 5 (11 %), un traitement inapproprié. La plupart des traitements inappropriés étaient dus à des tachyarythmies auriculaires.

**Conclusions:**

D’après notre analyse, la dysfonction associée au VDs est le facteur de risque le plus fréquemment mentionné en cas d’utilisation appropriée d’un DCI. Traiter efficacement les tachyarythmies auriculaires demeure une priorité. Des études de plus grande envergure s’imposent pour établir et valider le calcul du risque dans cette population.

Survival in adults with congenital heart disease (ACHD) has improved significantly with advances in surgery, intervention, and device therapy.^1^ However, sudden cardiac death (SCD) remains a leading cause of late mortality.^2^^,^^3^ Malignant ventricular arrhythmias are considered to be the main cause of SCD. Thus, implantable cardioverter-defibrillators (ICDs) are increasingly being considered in this growing patient group. A recent meta-analysis has shown very high rates of both appropriate and inappropriate shocks in this population, regardless of ICD indication (primary or secondary prevention).^4^ It additionally reported a high incidence of ICD-associated complications highlighting the importance of carefully evaluating the potential benefits vs risks and the importance of shared decision-making with patients.^4^

Patients with a systemic right ventricle (sRV) in a biventricular circulation are known to have a particularly high SCD risk.^5^ Dextro-transposition of the great arteries (d-TGA), with previous atrial switch repair, and congenitally corrected TGA (cc-TGA) are 2 of the most common defects with an sRV.^6^ Patients with an sRV tend to be evaluated for an ICD at a younger age than those with acquired heart disease and are therefore exposed to the risk of device-related complications and ICD shocks over a longer duration, potentially many decades. Current guidelines issue a weak recommendation to consider primary prevention ICD implantation in patients with severe sRV dysfunction.^7^, ^8^, ^9^ This recommendation gains strength if the sRV dysfunction is associated with nonsustained ventricular tachycardia (NSVT), unexplained syncope, functional impairment, a wide QRS, or severe systemic atrioventricular valve regurgitation.^7^

This systematic review aims to collate all literature on primary prevention ICD implantation in patients with an sRV due to TGA. The primary objectives are to ascertain (1) rate of appropriate ICD therapy (including shocks and antitachycardia pacing [ATP]) and (2) factors associated with appropriate ICD therapy in patients with primary prevention ICDs. The secondary objectives are to assess (1) rates of inappropriate therapies (ICD therapies for anything other than ventricular tachycardia [VT] or ventricular fibrillation [VF]) and (2) ICD complications. Ultimately, we aim to ascertain whether selection criteria for primary prevention ICDs in this population can be refined.

## Methods

This systematic review was conducted according to the Preferred Reporting Items for Systematic Reviews and Meta-Analyses (PRISMA) guidelines.^10^ The study protocol was registered on the PROSPERO international prospective register of systematic reviews (ID: CRD42023399892).

### Search strategy

The search was conducted in MEDLINE (Ovid) and Embase (Ovid) by the authors (JVY and XC-S) and informed by discussion with a librarian regarding keyword search creation. The search conducted included all articles from the inception of the above databases until the search date February 14, 2023. Detailed search strategy and search terms are outlined in Supplemental Appendix S1. References lists of the selected articles were also reviewed for additional relevant articles.

### Eligibility criteria

The study included randomized controlled trials, prospective and retrospective observational studies reporting the rate of ICD therapies, and associated clinical characteristics in patients with an sRV due to TGA who had an ICD implanted for primary prevention of SCD. There was no limit placed on the year of publication.

Patients had either d-TGA or cc-TGA. There was no prespecified age limitation. All ICD systems (single- and dual-chamber, with and without cardiac resynchronization therapy [CRT] or conduction system pacing) with any type of leads (endocardial, epicardial, and subcutaneous) regardless of manufacturer were included.

When available in the identified publications, the group of interest were compared with patients with an sRV due to TGA and primary prevention ICD without any appropriate shocks recorded.

Case reports, small case series with less than 5 patients, and studies including other congenital heart diseases, where specific subgroup analysis for patients with an sRV was not performed, were excluded. Systematic reviews and narrative reviews were excluded but used for manual review of reference lists. Studies written in a language other than English, with no available translation, and conference abstracts were also excluded.

### Selection of articles

The selection process was performed using the Covidence Systematic review software.^11^ After removing duplicated articles, one of 2 pairs of 2 reviewers (JVY and KA or JVY and XC-S) independently screened all articles by title and abstract. Conflicts were resolved through discussion between the 2 reviewers. When there was a disagreement, consensus was obtained with the help of the third reviewer (KA or XC-S). Full texts were then reviewed by 2 reviewers (JVY and XC-S). Disagreement at this stage was resolved by discussion between the 2 reviewers.

### Data extraction

Data extraction was performed by 2 investigators (JVY and XC-S) using a prespecified data collection proforma designed by the authors that included study design, baseline characteristics, outcomes, and predictors of outcomes. The primary outcomes examined were the rate of appropriate ICD therapy and predictors of appropriate ICD therapy in those with primary prevention ICDs. Secondary outcomes included inappropriate ICD therapy and device-related complications. Appropriate ICD therapy was defined as therapy (either shock or ATP) delivered to terminate VT and/or VF. Inappropriate ICD therapy was defined as therapy delivered for any other reason. Complications included periprocedural complications (occurring <30 days of implantation of the pulse generator or exchange procedure) and late complications (occurring >30 days after the procedure). Rates of appropriate and inappropriate ICD therapies were also assessed in those with secondary prevention ICDs.

Predictors of outcome were defined as any characteristic of the patient that was reported or included in the risk factor analysis.

### Quality assessment

Study quality and risk of bias were assessed by 2 independent reviewers (JVY and XC-S) based on the Newcastle Ottawa Quality Assessment scale for cohort studies.^12^ Conflicts were resolved through discussion between the 2 reviewers.

### Statistics

To assess whether rates of therapeutic shocks differed by appropriateness and reason for inappropriate shock(s), a Poisson model using all patients (including those with primary and secondary prevention ICDs) was used with the log of the number of patients as the intercept to enable comparison of rates. Sensitivity analyses were performed if any data points had substantially higher rates of shock than others, as this may bias results due to the sample size.

## Results

**Figure 1:**
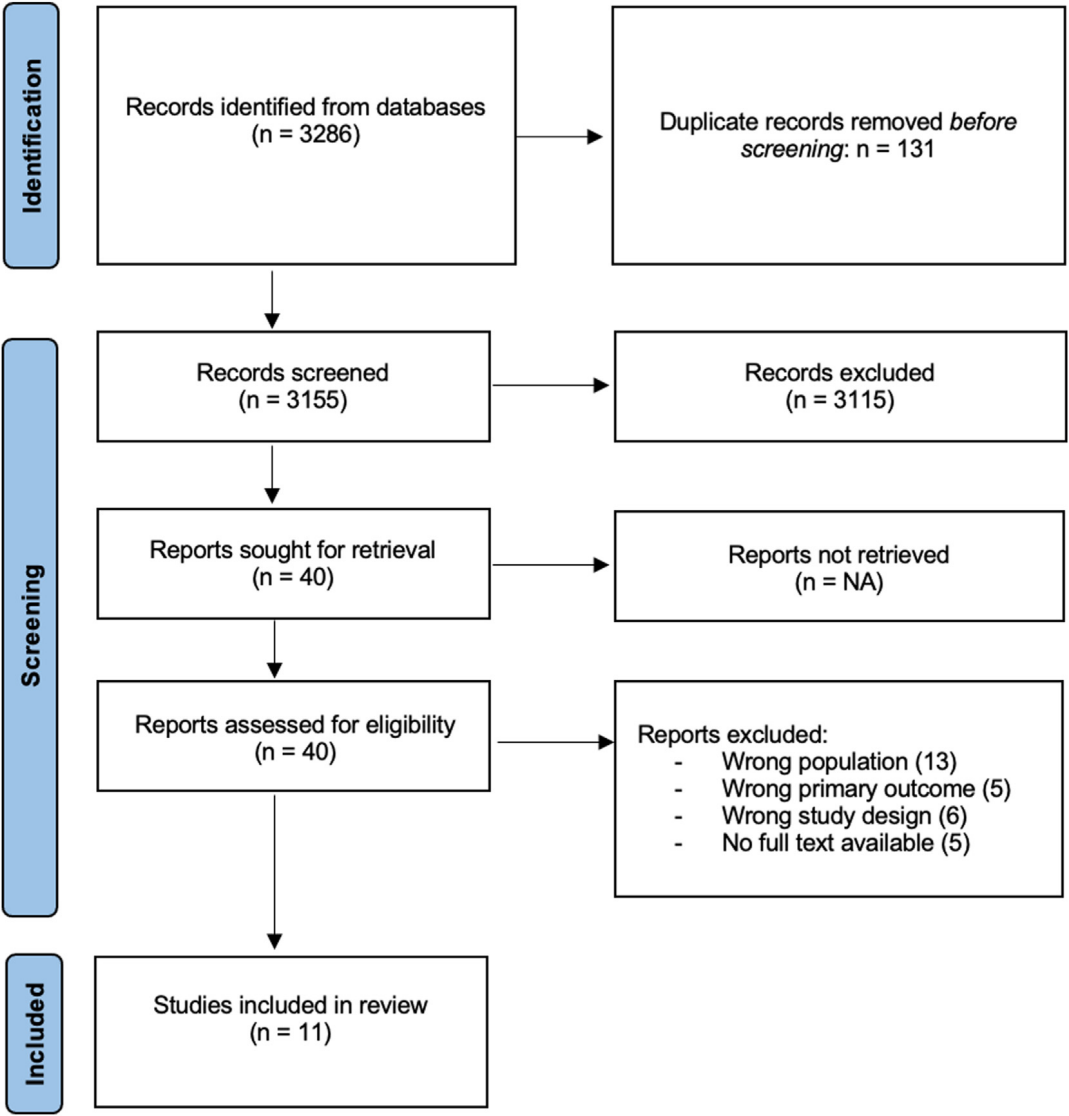
Preferred Reporting Items for Systematic Reviews and Meta- analyses (PRISMA) flowchart. The study selection process was conducted according to the PRISMA guidelines.^10^

Up to February 2023, a total of 3155 unique articles were identified. After screening titles and abstracts, 40 articles were retained. After reviewing full texts, 11 articles were selected for the systematic review. The full PRISMA flow diagram is shown in Figure 1.

### Study characteristics

**Figure 2:**
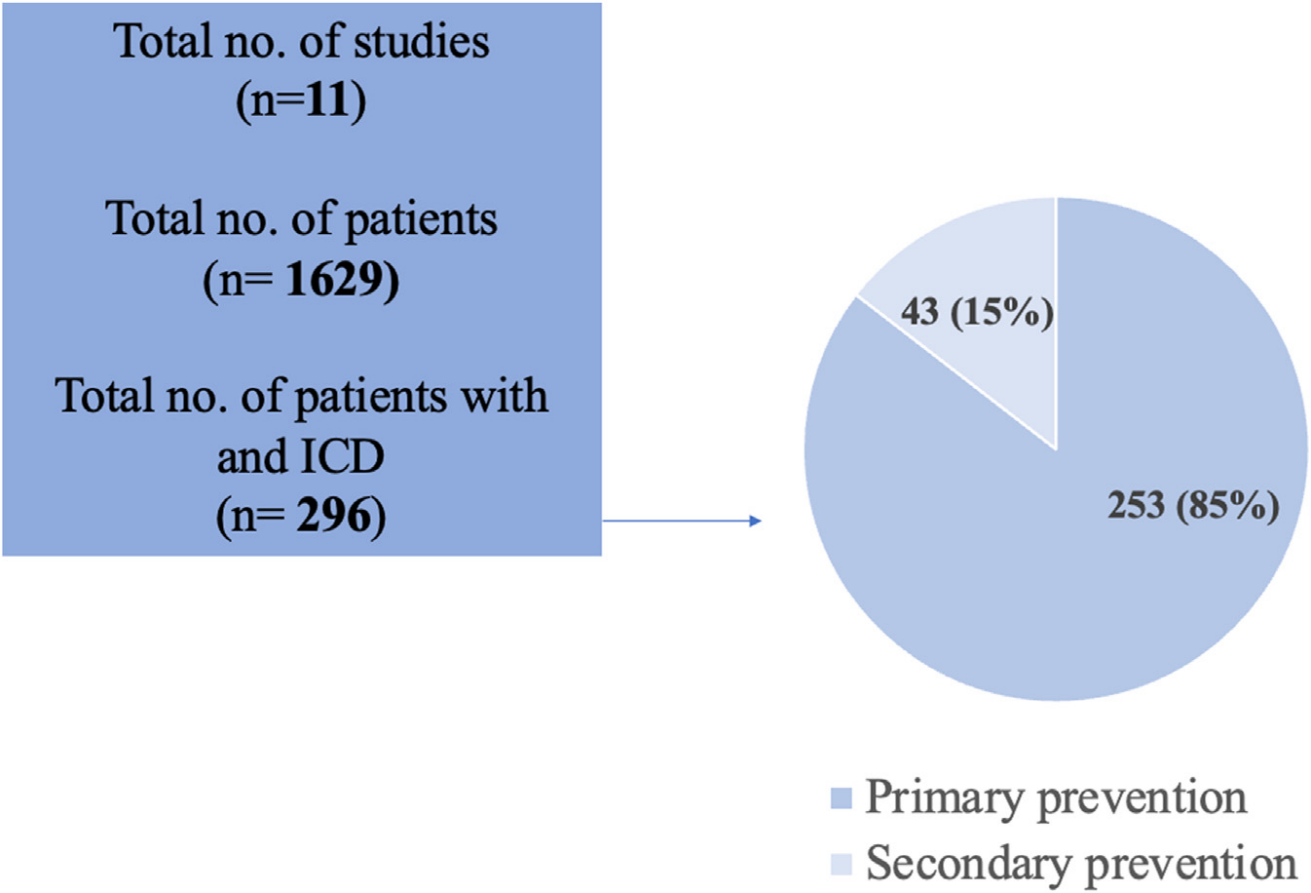
Patient population with an implantable cardioverter-defibrillator (ICD).

Selected studies for inclusion were published between 2008 and 2022 (Table 1). The majority were retrospective observational analyses with 1 case control study. Studies were conducted in Europe (n = 5, 46%), America (n = 4, 36%), and Australia (n = 2, 18%). The study cohort size ranged from small case series (n = 7) to large cohorts/registry-based studies (n = 1184). Among the 1629 patients included for analysis, 296 (18%) had an ICD, of whom most were for primary (n = 253, 85%) as compared with secondary (n = 43, 15%) prevention indications (Fig. 2). Most patients were male (69.5%). As individual patient level data were not included, we were unable to assess the entire cohort of 296 with respect to age or follow-up time. In the 4 studies that used parametric analysis, the average mean age was 31.7 years at the time of implantation. In the 7 studies that used nonparametric analysis, the average median age was 30.7 years at the time of ICD implantation. The mean or median follow-up time ranged from 19 months to 9.4 years. Of the 296 patients who had an ICD, 110 (37%) had a dual-chamber ICD, 23 (8%) had a single-chamber ICD, 17 (6%) had a CRD defibrillator, 2 (1%) had a subcutaneous ICD (S-ICD), and 144 (49%) patients had an ICD not otherwise specified.

**Table 1:** Characteristics of studies included in the systematic review

Author	Date, country	Study design	Follow-up time	Demographics—age at ICD implantation (y), sex (male), n (%)	Diagnoses (cc-TGA vs d-TGA)	No. of patients with primary prevention ICD	Appropriate therapy in primary prevention ICD	Complications in primary prevention ICD	Causes of inappropriate shocks	Predictors of shocks in primary prevention ICD	Appropriate shocks in secondary prevention ICD	Complications in secondary prevention ICD
Backhoff et al.^17^	2014, Germany	Single-centre retrospective case control study	3.5 y (range 5 mo to 7.2 y)	Median age 30.3, 33 (80)	41 d-TGA (100%)	12	1 ICD shock in 1 patient	4 inappropriate shocks in 3 patients, 1 lead failure, 2 ICD infection requiring change	3 shocks due to atrial arrhythmias, 1 due to failure of the Sprint Fidelis (Medtronic) lead	Multiple episodes of NSVT recorded by the device	N/A	N/A
Backhoff et al.∗^16^	2016, Germany	Multicentre retrospective cohort study	4.8 y (IQR: 2.5-8.5 y)	Median age 27.5 (IQR: 23.7-32.3), 28 (85)	33 d-TGA (100%)^18^	29	7 ICD therapies in 3 patients	12 inappropriate shocks in 8 patients, 5 lead dislodgement/failure, 2 ICD infection	10 shocks due to atrial arrhythmias, 2 due to failure of the Sprint Fidelis (Medtronic) lead	VT on Holter (2), impaired RV function (1)	No appropriate shocks	
Bouzeman et al.^15^	2014, France	Multicentre retrospective case series	19 mo (range 10-106 mo)	Median age 34 (range 28-40), 8 (67)	12 d-TGA (100%)	8	1 ATP without shock in 1 patient	1 inappropriate ATP in 1 patient, 1 lead dislodgement, 1 lead fracture	1 ATP due to atrial tachycardia		No appropriate shocks	1 died due to refractory cardiac arrest after defibrillation testing
Buber et al.^22^	2015, USA	Single-centre retrospective cohort study	4 y (range 1-9 y)	Median age 26 (range 13-41), 15 (83)	18 d-TGA (100%)	18	1 shock in 1 patient	33 inappropriate shocks in 10 patients, 3 lead fracture, 2 lead dislodgement, 2 recall, 1 undersensing, 1 endocarditis	5 patients had shocks due to atrial flutter, 3 patients had oversensing (Fidelis lead fracture), 1 had T-wave oversensing, 1 had SVT, 1 had sinus tachycardia	Moderate-severely reduced RV function, QRS 172 ms, NSVT on exercise test, NSVT on EPS	N/A	N/A
Grubb et al.^13^	2017, USA	Single-centre retrospective cohort study	139 patient-years	Mean age 25.9 ± 17.2, 43 (68)	34 d-TGA (54%), 29 cc-TGA (46%)	18	0	2 inappropriate shocks in 1 patient	2 shocks for atrial arrhythmias		4 (67%) shocks	0
Hohmann et al.^28^	2018, Germany	Single-centre retrospective and prospective cohort study	5 y (IQR: 2.2-15.1 y)	Mean age 31 ± 15, 8 (57)	7 d-TGA (50%), 7 cc-TGA (50%)	8	ICD therapies in 4 patients	7 inappropriate shocks in 4 patients	SVT, lead failure (unclear how many)		5 (83.3%) shocks	1 had inappropriate shocks
Kapa et al.^32^	2018, USA	Single-centre retrospective cohort study	7.2 ± 3.4 y	Mean age 42 ± 14, 81 (62.8)	129 cc-TGA	2	ICD therapy in 1 patient	Nil	N/A	RVEF <35% (HR: 6.090 [1.942-19.104], *P* = 0.0020), QRS duration (HR: 1.040 [1.021-1.060], *P* ≤ 0.0001)	2 (22%) ICD therapy	Nil
Khairy et al.^25^	2008, Canada	Multicentre retrospective cohort study	3.5 y (IQR: 1.5-5.5 y)	Mean age 28 ± 7.6, 33 (89.2)	37 d-TGA	23	1 shock in 1 patient	67 inappropriate shocks in 5 patients	Lead fracture, failure or oversensing, SVT, sinus tachycardia (unclear how many)	Severe systemic RV dysfunction, moderate TR, NSVT, secondary prevention indication, lack of β-blockers	4 (28.6%) patients had 18 shocks	25 inappropriate shocks in 4 (28.6%) patients
Ladouceur et al.^5^	2022, Europe	Multicentre retrospective cohort study	9.4 y (IQR: 4.9-12.9 y)	Median age 27.1 (19.9-34.9), 700 (59.1)	834 d-TGA (70.4%), 350 cc-TGA (29.6%)	121	8 shocks in 8 patients	14 patients had inappropriate shocks	11 shocks due to atrial arrhythmia, 3 due to lead dysfunction	Age (HR: 1.03 [1.01-1.05], *P* = 0.003), heart failure history (HR: 2.75 [1.47-5.16], *P* = 0.002), syncope (HR: 5.52 [2.47-12.34], *P* < 0.001), QRS duration (1.02 [1.01-1.03], *P* < 0.001), severe sRV dysfunction (HR: 3.06 [1.62-5.77], *P* < 0.001), LVOTO >36 mm (HR: 2.76 [1.56-4.90], *P* < 0.001)	N/A	N/A
Moore et al.^33^	2020, Australia	Single-centre retrospective cohort study	6.6 y (IQR: 3.3-11.5 y)	Median age 37 (IQR: 19), 35 (59)	7 d-TGA, 2 cc-TGA	9	3 shocks in 3 patients	N/A	Supraventricular arrhythmia, sinus tachycardia, T-wave oversensing, lead fracture (unclear how many)	Severe systemic ventricular impairment, NSVT, syncope	N/A	N/A
Wheeler et al.^14^	2013, Australia	Single-centre retrospective cohort study	4.6 y (range 1-5.4 y)	Median age 33 (29-38), 48 (53.9)	89 d-TGA (100%)	5	0	2 patients with inappropriate shocks	2 shocks for atrial arrhythmias	No predictor as no shocks		

ATP, antitachycardia pacing; cc-TGA, congenitally corrected transposition of the great arteries; d-TGA, dextro-transposition of the great arteries; EPS, electrophysiology studies; HR, hazard ratio; ICD, implantable cardioverter-defibrillator; IQR. interquartile range; LVOTO, left ventricular outflow tract obstruction; N/A, not applicable; NSVT, nonsustained ventricular tachycardia; RVEF, right ventricular ejection fraction; sRV, systemic right ventricle; SVT, sustained ventricular tachycardia; TR, tricuspid regurgitation; VT, ventricular tachycardia.

∗ Backhoff et al.^16^ may have included some patients who were included in the studied published by Backhoff et al. in 2014.^17^

The majority of studies (n = 7, 64%) reported patient factors associated with appropriate ICD therapy without conducting a dedicated risk factor analysis. Consequently, formal meta-regression analyses could not be performed to statistically assess factors associated with delivery of appropriate ICD therapies.

### Indications for primary prevention ICD

In the majority of patients, risk stratification for primary prevention ICD was based on several factors. The composite indications for primary prevention ICD implantation tended to include documented nonsustained ventricular arrhythmias, inducible VT/VF on electrophysiology studies, and severe sRV dysfunction (Table 2). Other indications included a composite of arrhythmia risk stratifiers, symptoms (presyncope, syncope, New York Heart Association II-III symptoms, and palpitations), electrocardiogram characteristics (prolonged QRS duration >140-180 ms), and documentation of atrial arrhythmias.

**Table 2:** Studies reporting on Primary prevention ICD insertion indications

Primary prevention indications	No. of studies that included this indication (n)	Indication as provided, total number of patients with each (n)
Systemic RV dysfunction	9	58
Severe RV dysfunction	5	28
RV dysfunction + heart failure	1	15
Moderate to severe RV dysfunction	1	9
Moderate RV dysfunction	1	1
Severe RV dysfunction + QRS >140 ms	1	3
RVEF <35%, LVEF <35%	1	2
Arrhythmias	7	69
Nonsustained ventricular arrhythmias	7	36
Inducible VT/VF on EPS∗	5	29
Atrial arrhythmia requiring medical therapy	1	4
Symptoms	4	35
Presyncope or syncope	3	18
Palpitations	1	12
Heart failure admission	1	5
Other	4	12
Severe subpulmonary LV dysfunction	2	2
QRS duration >180 ms	1	7
AV node ablation	1	3

Indications for primary prevention ICD were not detailed in all studies. Among the studies that did report indications, most reported more than 1 indication and some individual patients had more than 1 indication for primary prevention ICD implantation. Thus, the total number of patients captured by these indications does not equate to the total number of patients represented.

AV, atrioventricular; EPS, electrophysiology study; ICD, implantable cardioverter-defibrillator; LV, left ventricular; LVEF, left ventricular ejection fraction; LVOTO, left ventricular outflow tract obstruction; RV, right ventricle; RVEF, right ventricular ejection fraction; SVT, supraventricular tachycardia; VF, ventricular fibrillation; VT, ventricular tachycardia.

∗ Indications for EPS unable to be ascertained.

#### Primary prevention ICD

##### Primary outcomes

Appropriate ICD therapies were delivered in 23 of the 253 patients (9.1%) with a primary prevention ICD. Of these, 20 had appropriate ICD shocks and 3 had appropriate ATP. In studies that used parametric analysis (n = 1), the overall mean follow-up time was 7.2 years. In studies that used nonparametric analysis (n = 9), the overall average median follow-up time was 4.8 years. In 2 studies (n = 23),^13^^,^^14^ there were no appropriate ICD therapies delivered over a follow-up period of 139 patient-years in 1 study and 23 patient-years in the other study. Of the 23 patients who had appropriate ICD therapies, details regarding original indication for ICD implantation were only available for 9 patients. Four of the 9 patients had more than 1 indication for implantation. The most common reason for implantation was sRV dysfunction (n = 7), followed by NSVT (n = 3), VT on Holter (n = 2), NSVT on exercise (n = 1), NSVT on electrophysiology study (n = 1), prolonged QRS duration (n = 1), moderate tricuspid regurgitation (n = 1), and syncope (n = 1).

No information regarding inappropriate shocks was provided in the 23 patients who received appropriate shocks.

##### Secondary outcomes

In the 253 patients with a primary prevention ICD, 48 (19%) received 142 inappropriate shocks. Among those in whom the reason for inappropriate shocks was specified, majority were due to atrial arrhythmias (n = 34, 76%), followed by lead dysfunction (n = 10, 22%). In 1 patient (2%), inappropriate shock was due to sinus tachycardia (2%).

### Other complications after primary ICD implantation

Lead complications reported were 5 (2.0%) lead dislodgement/lead failures, 4 (1.6%) lead failures, 3 (1.2%) lead dislodgements, 4 (1.6%) lead fractures, and 5 (2.0%) ICD infections/endocarditis. There were no data regarding lead revisions or device extractions among this group.

#### Secondary prevention ICD

##### Outcomes for secondary prevention ICD

**Figure 3:**
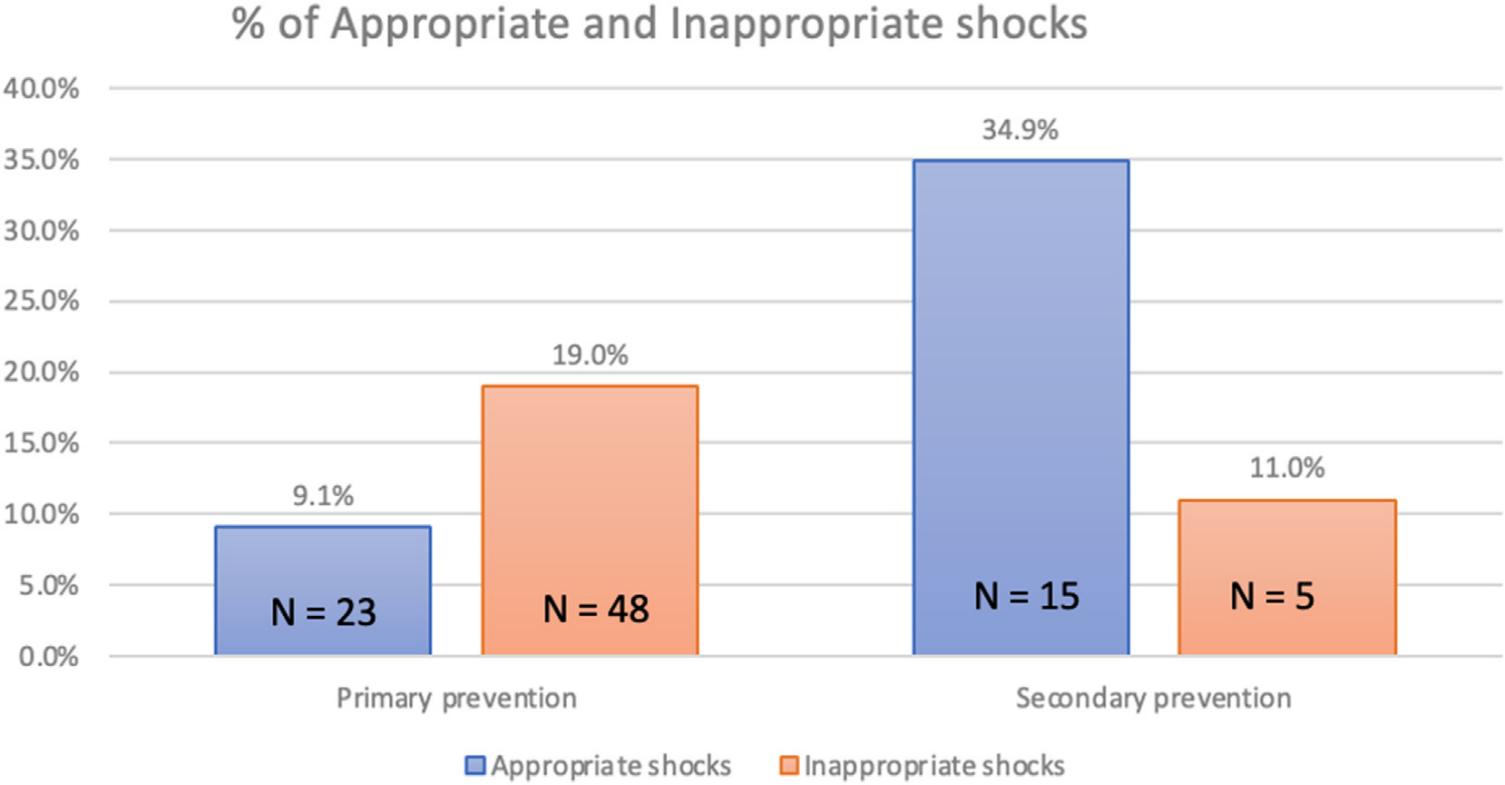
Percentage of appropriate and inappropriate shocks according to primary or secondary insertion indication.

In the 43 patients with a secondary prevention ICD, 15 (34.9%) received an appropriate and 5 (11%) an inappropriate ICD shock. In 1 patient, postimplantation defibrillation threshold testing resulted in refractory cardiac arrest and death.^15^ A comparison of the percentage of patients receiving appropriate vs inappropriate shocks, according to primary or secondary ICD indication, is shown in Figure 3. These demonstrate that the ratio of appropriate to inappropriate shock was 0.5:1 in the primary prevention and 3:1 in the secondary prevention cohorts identified by this systematic review. We were unable to break down the causes of inappropriate shocks in this group from the data provided.

### Statistics

#### Inappropriate shocks for supraventricular tachycardias in all patients

When information was available, data on the number of shocks split by indication, and the number of patients affected by these was extracted. Indications were classified as appropriate shocks, lead failure, and atrial arrhythmias, which included atrial tachycardia and SVT. Compared with those who had inappropriate shocks for atrial arrhythmias, appropriate shocks occurred at a significantly lower rate (β = –0.5198, *P* = 0.0354), and inappropriate shocks due to lead dysfunction also occurred at a lower rate (β = –0.7108, *P* = 0.0997) (Table 3).

**Table 3:** Results of Poisson models for rate of implantable cardioverter-defibrillator shocks, compared with inappropriate shocks for atrial arrhythmias, in all data from the study (left) and the sensitivity analysis excluding one especially high observation (right)

	All data			Sensitivity analysis		
	Coefficient	Standard error	*P* value	Coefficient	Standard error	*P* value
Appropriate shocks	–0.5198	0.2470	0.0354	–0.0408	0.2792	0.884
Lead failure	–0.7108	0.4292	0.0997	–0.2381	0.4485	0.605

One data point had an observably higher rate of shocks (28 shocks in 5 patients) for atrial arrhythmias.^5^^,^^13^^,^^14^^,^^16^^,^^17^ A sensitivity analysis was performed excluding this. After removing this, the standard errors for parameters in the Poisson model were wider, and coefficients were lower. Appropriate shocks occurred at a marginally higher rate, which was no longer statistically significant (β = –0.0408, *P* = 0.884), and the coefficient for lead failure shocks decreased (β = –0.2318, *P* = 0.605).

### Quality assessment of the studies

Quality evaluation was performed based on the original research question posed by each respective author, which was not necessarily the same as our research question. In these studies, the information required for our review was extracted from subgroup analyses.

Using the Newcastle Ottawa Quality Assessment scale, 7 studies were assessed as of good quality and 4 were of fair quality (Supplemental Table S1).

## Discussion

Although atrial switch procedures transformed early outcomes, late mortality from heart failure and atrial and ventricular arrhythmias have increased in prevalence with age.^18^^,^^19^ ACHD guidelines have consensus recommendations for both secondary (class I) and primary (class IIb) prevention ICD implant, with the latter predominantly for sRV dysfunction.^20^ Assessment of the efficacy and complications related to ICD therapy is of paramount importance in guiding appropriate implantation.

The study identified low rates of appropriate shocks in those with an sRV and primary prevention ICD. Although our primary objective was to assess those with primary prevention ICDs, we also found high rates of appropriate shocks in those with an sRV and secondary prevention ICDs. We demonstrate that approximately 1 in 10 of primary prevention sRV patients have appropriate ICD therapies over an average follow-up period of 7.2 years (mean) and 4.8 years (median), whereas approximately 1 in 3 secondary prevention sRV patients had appropriate shocks during the same follow-up period. In those with appropriate ICD therapies, sRV dysfunction was the most frequently reported reason for ICD implantation. This is consistent with other series assessing outcomes in patients with d-TGA. Broberg et al.^18^^,^^21^ studied 1168 patients, of whom 91 (8.8%) patients experienced the primary outcome (death, transplantation, or mechanical circulatory support). Moderate-severe RV systolic dysfunction was significantly associated with the primary outcome on both univariable (hazard ratio [HR]: 5.00 [95% confidence interval: 3.16-7.93], *P* = 0.001) and bivariable analyses. In a cohort of 1184 patients with sRVs, Ladouceur et al.^5^ also found that severe RV dysfunction was a risk factor for major adverse events such as ventricular arrhythmias (HR: 3.06 [95% confidence interval: 1.62-5.77], *P* < 0.001).

A large number of reported potential risk factors were identified in the present review including age at assessment, age at surgery, surgical era and technique, decompensated heart failure, syncope, NSVT, tricuspid regurgitation, left ventricular outflow tract obstruction, prolonged QRS duration, reduced subpulmonary left ventricular function, left ventricular hypertrophy, precapillary pulmonary hypertension, and lack of β-blocker therapy. More recent literature has raised potential new factors, such as RV end-diastolic pressure as a surrogate of an enlarged systemic RV as a future potential risk stratification strategy.^22^ Unfortunately, the small number of appropriate shocks in this review precluded statistical analyses on individual risk factors. Thus, we were unable to ascertain which risk factors predict appropriate ICD shocks.

A recent risk prediction model based on a large multicentre cohort has been developed.^5^ Although it confirms many of the risk factors used by the papers in this systematic review, patients with a primary prevention ICD represented only 10.2% of the derivation cohort, and most were classified as low-risk with their risk prediction model.^5^ Further validation of the risk model is thus needed for guidance for primary prevention ICD in sRV cohorts.

Appropriate to inappropriate shock ratios were 0.5:1 vs 3:1 in the primary vs secondary prevention groups, with appropriate shock rates of 9% and 35%, respectively. This most likely relates to better-defined risk factors in patients with secondary prevention ICDs and potential mismatch between risk factors and substrate in those with primary prevention ICDs. Lead fracture, lead dislodgement, and infection occurred in approximately 1 in 25 patients with ICD implants. In noncongenital cardiology cohorts, appropriate ICD therapies occur in up to 13% of patients over 2 years of follow-up,^23^ and appropriate to inappropriate shock ratios are observed to be around 1.2-3.5 to 1.^21^^,^^23^ Complications occur in around 9% of patients over a 16-month follow-up period.^24^ Most inappropriate shocks in our cohort were due to supraventricular tachycardias including atrial flutter and atrial tachycardias. Although shocks for atrial tachyarrhythmias in this population are technically classified as inappropriate, significant SCD risk exists with fast ventricular conduction due to decreased stroke volume and sRV myocardial oxygen supply to demand mismatch.^25^^,^^26^ Furthermore, Khairy et al.^27^ found that among those with appropriate shocks, supraventricular tachyarrhythmias were found to precede or coexist with ventricular tachyarrhythmias in 50% of patients. This adds to the challenges in interpreting the literature, raising an intriguing possibility that some inappropriate shocks may in fact be life-saving in the sRV population. Considering that a subset of the rapidly conducted atrial flutter episodes treated by defibrillation may have subsequently degenerated to malignant ventricular arrhythmia, many could ostensibly be relabelled appropriate. This would favourably impact the proportion of appropriate to inappropriate shocks. Better medical therapy and earlier ablation strategies may relieve the burden of such “inappropriate” shocks for atrial arrhythmia.

Use of β-blockers was examined in 6 of the 11 studies.^5^^,^^14^^,^^17^^,^^22^^,^^27^^,^^28^ Ladouceur et al.^5^ found that β-blocker use was significantly associated with SCD in patients with d-TGA on univariate analysis (HR: 2.59 [1.23-5.48], *P* = 0.012), which was no longer significant on multivariate analysis. Wheeler et al.^14^ found no association between β-blocker use and SCD. Khairy et al.^27^ found that lack of β-blockers was predictive of appropriate ICD shocks in both the univariable analysis (HR: 11.3 [1.3, 100.1], *P* = 0.0303) and the multivariable analysis (HR: 16.7 [1.3, 185.2], *P* = 0.0301). They proposed that β-blockers, in addition to having beneficial effects on primary ventricular arrhythmias, could potentially reduce the risk of degeneration of supraventricular arrhythmias in fatal events, by modulating atrioventricular nodal effective refractory period, augmenting right ventricular diastolic filling, reducing sRV dysfunction, and reducing subendocardial ischemia. Furthermore, β-blockers have been associated with positive RV remodelling in patients with an sRV.^27^

There are important anatomic considerations when considering ICD implant in this population. These include the presence of baffle leaks or obstructions, venous access occlusions, accessibility of the coronary sinus, and the presence of anatomic variability such as left-sided superior vena. The S-ICD may be an attractive alternative in selected patients.^29^ However, the S-ICD does not provide antibradycardia pacing, which is problematic due to a high prevalence of sinus node dysfunction (d-TGA) or AV block (cc-TGA). Moreover, S-ICD is not capable of delivering ATP to address monomorphic substrate-related VTs, or CRT, which has been shown to improve QRS duration and functional status in those with an sRV and pacing-induced or (in selected patients) intrinsic dyssynchrony and progressive heart failure.^30^ In a study assessing S-ICD in young ACHD, 2.8% of the study population required change to/addition of transvenous pacing systems due to the development of bradycardia/need for CRT.^31^ Moreover, the complex anatomy can affect sensing vectors, which can potentially render patients ineligible for an S-ICD or at increased risk of inappropriate shocks. There are currently limited studies regarding the use of S-ICDs in patients with ACHD, particularly in those with sRV anatomy.

### Limitations

This review reveals a paucity of comprehensive patient risk factors and follow-up data to guide primary prevention ICD implantation in CHD patients with an sRV. Currently available publications are limited by cohort size with low absolute event rates and at large observational design. It is important to note that although the overall quality of the studies is fairly good, the lack of statistical power in directly addressing the specific research question of this systematic review remains a limitation. Lack of detail regarding individual patients and shocks rendered data extraction and analyses challenging. Furthermore, lack of detail in papers limits the description of device data (ie, transvenous vs epicardial systems, single- vs dual-chamber devices, and ICD programming details) and concomitant therapy indications (ie, pacemaker or CRT indications). Delivered shocks were stratified as appropriate or inappropriate as per original literature. It is not possible to be certain that there was uniformity across the cohorts. Most studies focussed on outcomes of patients with an sRV and an ICD, rather than identifying predictors of appropriate ICD therapies. Collaborative multicentre research is required to further understand this population to guide optimal ICD implantation in this cohort.

## Conclusions

This review reveals the need for wider population-based validated data to guide primary prevention ICD implantation in patients with sRV. Our systematic review identified that approximately 1 in every 10 patients with an sRV and a primary prevention ICD experiences an appropriate ICD shock within 5-7 years of follow-up. However, this rate may be higher if therapies for fast conducted atrial arrhythmias, which may be poorly haemodynamically tolerated, were deemed appropriate. The rates of lead-related and infection complications are not negligible. Challenges in ascertaining those most at risk remain, although sRV dysfunction emerges as the most consistently reported risk factor. This review supports current approaches to ICD implantation in patients with an sRV but highlights the need for larger scale studies, with prospective, multiparametric SCD risk factor stratification, to develop and validate improved risk calculation on a patient-tailored level. In addition, the need to pursue effective treatment of atrial arrhythmias in those with an ICD remains a priority.

## References

[1] Kella D.K., Merchant F.M., Veledar E., Book W., Lloyd M.S. (2014). Lesion-specific differences for implantable cardioverter defibrillator therapies in adults with congenital heart disease. Pacing Clin Electrophysiol.

[2] Oechslin E.N., Harrison D.A., Connelly M.S., Webb G.D., Siu S.C. (2000). Mode of death in adults with congenital heart disease. Am J Cardiol.

[3] Naidu P., Grigg L., Zentner D. (2017). Mortality in adults with congenital heart disease. Int J Cardiol.

[4] Vehmeijer J.T., Brouwer T.F., Limpens J. (2016). Implantable cardioverter-defibrillators in adults with congenital heart disease: a systematic review and meta-analysis. Eur Heart J.

[5] Ladouceur M., Van De Bruaene A., Kauling R. (2022). A new score for life-threatening ventricular arrhythmias and sudden cardiac death in adults with transposition of the great arteries and a systemic right ventricle. Eur Heart J.

[6] Ladouceur M., Waldmann V., Bartoletti S., Chaix M.-A., Khairy P. (2023). Ventricular arrhythmia in congenital heart diseases with a systemic right ventricle. Int J Cardiol Congenit Heart Dis.

[7] Khairy P., Van Hare G.F., Balaji S. (2014). PACES/HRS Expert Consensus Statement on the Recognition and Management of Arrhythmias in Adult Congenital Heart Disease: developed in partnership between the Pediatric and Congenital Electrophysiology Society (PACES) and the Heart Rhythm Society (HRS). Endorsed by the governing bodies of PACES, HRS, the American College of Cardiology (ACC), the American Heart Association (AHA), the European Heart Rhythm Association (EHRA), the Canadian Heart Rhythm Society (CHRS), and the International Society for Adult Congenital Heart Disease (ISACHD). Heart Rhythm.

[8] Priori S.G., Blomstrom-Lundqvist C., Mazzanti A. (2015). 2015 ESC Guidelines for the management of patients with ventricular arrhythmias and the prevention of sudden cardiac death: the Task Force for the Management of Patients with Ventricular Arrhythmias and the Prevention of Sudden Cardiac Death of the European Society of Cardiology (ESC). Endorsed by: Association for European Paediatric and Congenital Cardiology (AEPC). Eur Heart J.

[9] Zeppenfeld K., Tfelt-Hansen J., de Riva M. (2022). 2022 ESC Guidelines for the management of patients with ventricular arrhythmias and the prevention of sudden cardiac death: developed by the task force for the management of patients with ventricular arrhythmias and the prevention of sudden cardiac death of the European Society of Cardiology (ESC) Endorsed by the Association for European Paediatric and Congenital Cardiology (AEPC). Eur Heart J.

[10] Page M.J., McKenzie J.E., Bossuyt P.M. (2021). The PRISMA 2020 statement: an updated guideline for reporting systematic reviews. Syst Rev.

[11] Covidence systematic review software. https://www.covidence.org.

[12] Wells G., Shea B., O’Connell D., Peterson J., Welch V. (2011). The Newcastle-Ottawa Scale (NOS) for assessing the quality of case-control studies in meta-analyses. Eur J Epidemiol.

[13] Grubb A.F., Shah G., Aziz P.F., Krasuski R.A. (2017). Pacemaker and defibrillator implantation in patients with transposition of the great arteries. J Innov Card Rhythm Manag.

[14] Wheeler M., Grigg L., Zentner D. (2014). Can we predict sudden cardiac death in long-term survivors of atrial switch surgery for transposition of the great arteries?. Congenit Heart Dis.

[15] Bouzeman A., Marijon E., de Guillebon M. (2014). Implantable cardiac defibrillator among adults with transposition of the great arteries and atrial switch operation: case series and review of literature. Int J Cardiol.

[16] Backhoff D., Kerst G., Peters A. (2016). Internal cardioverter defibrillator indications and therapies after atrial baffle procedure for d-transposition of the great arteries: a multicenter analysis. Pacing Clin Electrophysiol.

[17] Backhoff D., Müller M., Ruschewski W., Paul T., Krause U. (2014). ICD therapy for primary prevention of sudden cardiac death after Mustard repair for d-transposition of the great arteries. Clin Res Cardiol.

[18] Broberg C.S., van Dissel Alexandra C., Minnier J. (2022). Long-term outcomes after atrial switch operation for transposition of the great arteries. J Am Coll Cardiol.

[19] van Dissel A.C., Opotowsky A.R., Burchill L.J. (2023). End-stage heart failure in congenitally corrected transposition of the great arteries: a multicentre study. Eur Heart J.

[20] Baumgartner H., De Backer J., Babu-Narayan S.V. (2021). 2020 ESC Guidelines for the management of adult congenital heart disease: the Task Force for the management of adult congenital heart disease of the European Society of Cardiology (ESC). Endorsed by: Association for European Paediatric and Congenital Cardiology (AEPC), International Society for Adult Congenital Heart Disease (ISACHD). Eur Heart J.

[21] Moss Arthur J., Schuger C., Beck Christopher A. (2012). Reduction in inappropriate therapy and mortality through ICD programming. N Engl J Med.

[22] Buber J., Ackley T.J., Daniels C.J. (2016). Outcomes following the implantation of cardioverter-defibrillator for primary prevention in transposition of the great arteries after intra-atrial baffle repair: a single-centre experience. Europace.

[23] Dichtl W., De Sousa J., Rubin Lopez J.M. (2023). Low rates of inappropriate shocks in contemporary real-world implantable cardioverter defibrillator patients: the CARAT observational study. Europace.

[24] Ezzat V.A., Lee V., Ahsan S. (2015). A systematic review of ICD complications in randomised controlled trials versus registries: is our ‘real-world’ data an underestimation?. Open Heart.

[25] Khairy P. (2017). Sudden cardiac death in transposition of the great arteries with a Mustard or Senning baffle: the myocardial ischemia hypothesis. Curr Opin Cardiol.

[26] Khairy P., Silka M.J., Moore J.P. (2022). Sudden cardiac death in congenital heart disease. Eur Heart J.

[27] Khairy P., Harris L., Landzberg M.J. (2008). Sudden death and defibrillators in transposition of the great arteries with intra-atrial baffles: a multicenter study. Circ Arrhythm Electrophysiol.

[28] Hohmann S., Duncker D., König T. (2018). Implantable cardioverter defibrillator therapy in grown-up patients with transposition of the great arteries-role of anti-tachycardia pacing. J Thorac Dis.

[29] D’Souza B.A., Epstein A.E., Garcia F.C. (2016). Outcomes in patients with congenital heart disease receiving the subcutaneous implantable-cardioverter defibrillator: results from a pooled analysis from the IDE Study and the EFFORTLESS S-ICD Registry. JACC Clin Electrophysiol.

[30] Kharbanda R.K., Moore J.P., Lloyd M.S. (2022). Cardiac resynchronization therapy for adult patients with a failing systemic right ventricle: a multicenter study. J Am Heart Assoc.

[31] Silvetti M.S., Bruyndonckx L., Maltret A. (2023). The SIDECAR project: S-IcD registry in European paediatriC and young Adult patients with congenital heaRt defects. Europace.

[32] Kapa S., Vaidya V., Hodge D.O. (2018). Right ventricular dysfunction in congenitally corrected transposition of the great arteries and risk of ventricular tachyarrhythmia and sudden death. Int J Cardiol.

[33] Moore B.M., Cao J., Cordina R.L., McGuire M.A., Celermajer D.S. (2020). Defibrillators in adult congenital heart disease: long-term risk of appropriate shocks, inappropriate shocks, and complications. Pacing Clin Electrophysiol.

