# Supplementary material for: Implantable Cardioverter-Defibrillator Therapy in Patients With Transposition of the Great Arteries: A Systematic Review of the Literature
Source: CJC Pediatr Congenit Heart Dis. 2025 Apr 24;4(5):249–59. doi: 10.1016/j.cjcpc.2025.04.006 (PMC12835910; doi:10.1016/j.cjcpc.2025.04.006)
Supplement: Supplementary Material [file mmc1.pdf]

Supplementary Material

Supplemental Appendix S1:
The following MESH terms and keywords were used in the search strategy: ‘Mustard’, ‘Senning’, ‘atrial switch’, ‘dtga’, ‘d-tga’, ‘d-transposition’, ‘cctga’, ‘cc-tga’, ‘l-transposition’, ‘ltga’, ‘l-tga’, ‘congenitally corrected transposition’, ‘transposition of the great arteries’, ‘systemic rv’, ‘systemic right ventricle’, ‘defibrillator’, ‘icd’, ‘aicd’, ‘automatic implantable defibrillator’, ‘implantable cardioverter defibrillator’, ‘device’.

Supplemental Table S1: Newcastle Ottawa Quality Assessment scale results

	**Selection**	**Comparability**	**Outcome**	**Study Quality**
Backhoff, 2014	4	0	2	Fair
Backhoff, 2016	4	0	2	Fair
Bouzeman, 2014	4	0	2	Fair
Buber, 2015	4	1	3	Good
Grubb, 2017	4	1	3	Good
Hohman, 2018	3	1	3	Good
Khairy, 2008	4	2	2	Good
Moore, 2020	4	1	2	Good
Kapa, 2018	4	1	2	Good
Ladouceur, 2022	4	1	2	Good
Wheeler, 2013	4	0	2	Fair
